# Civil Malpractice

**Published:** 1877-09

**Authors:** 


### Books Reviewed.

---

*Civil Malpractice: A Treatise on Surgical Jurisprudence, with Chapters on Skill in Diagnosis and Treatment, Prognosis in Fractures, and on Negligence.* By MILO A. McCLELLAND, M. D. New York: Published by Hurd & Houghton, 1877. Price $3.50.

We cannot do better than quote the author's definitions of malpractice and of a "quack," as given in Chapter I.

"Malpractice may be divided into three kinds, namely: 1. 'Ethical Malpractice,' in which persons claiming to be medical men bring suits against physicians or against Medical Societies, for alleged insults to their professional dignity. 2. 'Civil Malpractice,' in which patients bring suits for damages, which they have or think they have sustained through want of skill, or negligence on the part of their attending physician. 3. 'Criminal Malpractice,' in which the people or State is made the plaintiff.

"Suits under the first classification are usually instituted by 'quacks.' This term, in the medical profession, is applied to any one, whether he has any professional education or not, who violates §§ 3, 4, Art. I, Ch. II., of what is known as the Code of Ethics of the American Medical Association, which declares it 'to be derogatory to the dignity of the profession to resort to public advertisements, or private cards, or hand-bills, inviting the attention of individuals affected with particular diseases; *publicly offering advice and medicine to the poor, gratis; or promising radical cures; or to publish cases and operations in the daily prints, or to suffer such publications to be made,* (the italics are our own. ED.) to invite laymen to be present at operations, to boast of cures and remedies, to adduce certificates of skill and success, or to perform any other similar acts. These are the ordinary practices of empirics, and are highly reprehensible in a regular physician. Equally derogatory to professional character is it for a physician to hold a patent for any surgical instrument or medicine; or to dispense a secret *nostrum*, whether it be the composition or exclusive property of himself or of others. For, if such nostrum be of real efficacy, any concealment regarding it is inconsistent with beneficence and professional liberality, and if mystery alone gives it value and importance, such craft implies either disgraceful ignorance or fraudulent avarice. It is also reprehensible for physicians to give certificates attesting the efficacy of patent or secret medicines, or in any way promote the use of them.'

"So, also, the term is applied to those practitioners of the 'Specific School,' those 'transcendental pathologists,' who substitute for anatomy, physiology, and common sense, a dogma, upon which to build an enlightened experience, who consider as signs of the same disease, and as possessing equal value, the following symptoms: 'Insatiable thirst, pallor of the face, scrofula, sweating of the head after sleep, burning in the palms of the hands, attacks of suffocation, furuncles, vomiting of blood, hiccough after eating or drinking, cutting pain in the rectum while at stools, absence of venereal desire, unbridled lusts, somnolency during the day after eating, paroxysms of anger bordering on mental alienation, tears frequently at the slightest causes,' &c., &c.; and who assure us in their therapeutics, that the administration of the 1,000,000,000,000,000,000,000th of a grain of carbonate of lime—common chalk—produces no less than a thousand and ninety symptoms, from which I select the following: 'On the fifth day, itching on the border of the eyelids; thirteenth day, *in the evening*, on going out, unsteady gait; seventeenth day, ardent venereal desires, especially during a walk *before dinner*; twenty-fiirst day, great heat at the extremity of the big toe; twenty-eighth day, itching at the anterior part of the glans penis, after urination,' * and so on, *ad nauseam.*

"As this volume may fall into the hands of some who are not familiar with the assumptions of these refined Pathologists and Therapeutists, I will be pardoned for presenting a few more flowers culled from their choicest conservatory, Jahr's Manual of Medicine, 'translated from the German by authority of the North American Academy of the Homœopathic Healing Art.' As the result of the attenuations and shakings certain of the remedies receive —and I may say here, most of them are taken from the regular arsenal—we have, 'Drawing pain in *hollow* teeth, extending to the eyebrows; cracked *upper* lip; stitches in *hollow* teeth, *when biting*; pain and pungency in the *elbow*, which does not allow one to stretch or exert the arm; pungency in the knee and bend of the knee; inflammation and swelling of *one-half* of the nose (which side the record saith not); torpor and stiffness of one-*half* of the tongue (probably the same side as of nose); blood blisters on the *inside* of the *upper* lip; loss of appetite, chiefly for bread and tobacco-smoking; rending and stinging in corns; red itching spots on the *shin-bone*; tingling in the *points* of the toes; perspiration on the hands, and *between* the fingers; stitches in the ankle *when stepping out*; a voluptuous tickling on the *sole* of the foot, *after scratching a little*, making a *man* (used in the sense of totality,—men, women, children?) almost *mad*; ulceration of *big* toe, with a prickling pain; an itching, tickling sensation at the *outer* edge of the *palm* of the *left* hand, which obliges the person to scratch.'

"Chamomile is followed by the following *mental* effects: 'Hypochondriac paroxysms of anxiety, as if the heart would break; restlessness, with anxious groaning and tossing about; irritable readiness to weep, with *whining* and *howling*, frequently on account of *old* or imaginary offences.'

"Chloride of sodium—common salt—is followed by more disastrous effects: 'Melancholic sadness, with searching for many *unpleasant* things; much weeping, and *increased* by consolation; sorrowfulness about futurity; anxiousness, also *during* a thunder-storm, *chiefly* at night; indolence, aversion to talk, joylessness, hasty impatience, and irritability; easily frightened; hate of *former* offenders; inclination to laugh; great weakness of memory and forgetfulness; awkwardness,' &c., &c.

"Sulphur is no better in its mental influence, if anything worse. Its administration is followed by 'sadness and dejection: melancholy, with doubts about his soul's welfare (suggestive); great inclination to weep frequently, alternating with laughing; inconsolableness, and reproaches of conscience about every action; attacks of anxiety, *in the evening*; nocturnal fear of spectres; philosophical and religious reveries; insanity, with imagination as if he were in possession of beautiful things and in abundance of everything.' But enough of this drivelling nonsense. What else can we call them but 'quacks'—ignorant pretenders to knowledge they do not possess?"

Would not many of the profession find it well to consider this extract carefully, and follow its teachings in spirit as well as in form?

* Hahnemann, Treatise on Chronic Diseases.

The greater part of the work consists of cases cited from various sources to illustrate all probable or possible ground upon which a suit for malpractice can be founded. The leading testimony, charges and the judgments of all the noted cases in the various States and England are given when of interest; and then the author gives his own views and also any remarks which he could find bearing on the subject.

It is well worth reading by every surgeon or physician, since the opinions of the author are sound and to be relied upon. C.

